# Positive surgical margins may not affect the survival of patients with renal cell carcinoma after partial nephrectomy: A meta-analysis based on 39 studies

**DOI:** 10.3389/fonc.2022.945166

**Published:** 2022-08-10

**Authors:** Renran Bai, Liang Gao, Jiawu Wang, Qing Jiang

**Affiliations:** ^1^ Department of Nephrology, Qianjiang Central Hospital, Chongqing, China; ^2^ Department of Urology, the Second Affiliated Hospital of Chongqing Medical University, Chongqing, China

**Keywords:** partial nephrectomy, positive surgical margin, prognosis, renal cell carcinoma, survival

## Abstract

**Background:**

So far, whether positive surgical margin(PSM) has adverse effects on the prognosis of patients is still controversial, so we designed this study to systematically evaluate the effect of PSM on the prognosis of patients with renal cell carcinoma (RCC) after partial nephrectomy (PN).

**Methods:**

On the basis of three electronic databases (PubMed, Embase and the Cochrane Library) up to May 2022, all case–control studies (CCSs) comparing the effects of PSM and negative surgical margin (NSM) after PN on the oncological results of RCC patients were included. Two evaluators independently conducted a systematic literature search and extracted the data we needed. The methodological quality of all studies was evaluated by the modified Newcastle–Ottawa scale. The odds ratio (OR) was used to describe the results for dichotomous variables, and the meta-analysis was conducted using Cochrane Review Manager 5.2 and Stata 14.2.

**Results:**

A total of 39 studies involving 21461 patients were included in our meta-analysis. The pooled results showed that the rates of tumor recurrence (OR 3.93, 95% CI 2.95-5.24; p < 0.00001) and metastasis (OR 4.63, 95% CI 3.11-6.88; p < 0.00001) in the PSM group were significantly higher than those in the NSM group. However, there were no significant differences in the rates of all-cause death (OR 1.35, 95% CI 0.92-1.99; p = 0.13) or cancer-specific death (OR 0.99, 95% CI 0.51-1.94; p = 0.99) between the two groups. In addition, subgroup analyses were carried out according to different average follow-ups, which revealed similar results.

**Conclusion:**

Insignificant differences in survival between the PSM and NSM groups were observed, although significant differences in recurrence and metastasis in the PSM group were reported. Our study supported that close monitoring might be another effective choice for patients with PSM after PN. Considering the possible limitations, we recommended cautious interpretation of our results.

## 1 Introduction

Partial nephrectomy (PN) is one of the standard treatments for renal cell carcinoma (RCC) (1), and it has been shown to be as safe and effective as radical nephrectomy (RN) for selected patients (2, 3). According to the surgeons’ preference along with the feasibility of technology, a robot-assisted, pure laparoscopic or open method can be chosen for PN. Theoretically, the purpose of the operation is to completely remove the tumor and provide a negative surgical margin (NSM) while preserving as much normal renal parenchyma as possible (4). However, positive surgical margins (PSM) occasionally occur, with an incidence of 2-8% (5), which is defined as the presence of cancer cells at the parenchymal inked margin of resection.

Dozens of studies have shown that PSM is an independent predictor for progression-free survival (PFS) due to significantly more long-term recurrences (6). However, other studies have shown a negative correlation between PSM and disease progression or death (7, 8). To date, whether PSM has an adverse effect on the prognosis of patients is still controversial (9). Therefore, we carried out a systematic review and meta-analysis to explore the effects of PSM on patients with RCC after PN.

## 2 Materials and methods

### 2.1 Search strategy

Two reviewers independently and systematically searched the literature published in three electronic databases (PubMed, Embase and the Cochrane Library) prior to May 2022. The search terms included recurrence/death/metastasis/oncologic outcome/survival AND surgical margin AND nephron-sparing surgery/partial nephrectomy. In addition, the reference lists from identified publications were also searched.

### 2.2 Inclusion criteria and data extraction

This study followed the Preferred Reporting Items for Systematic Reviews and Meta-Analyses (PRISMA) statement. Because of the nature of our study, randomized controlled trials (RCT) and prospective cohort studies were unethical. Therefore, all case–control studies (CCSs) comparing the effects of PSM and NSM after partial nephrectomy on the oncological results in RCC patients were included. When articles were not written in English, they were translated for data extraction if possible.

After reviewing the title and abstract, we reviewed the full text to more fully assess whether it met the inclusion criteria: the population we included in the study comprised patients who underwent partial nephrectomy with all surgical methods (open surgery, laparoscopy, robot-assisted) and whose pathological diagnosis was renal cell carcinoma (including any histological subtype). We excluded studies that approved the treatment of renal cell carcinoma through ablation therapy and excluded studies on the pathological diagnosis of benign tumors after partial nephrectomy. Studies without available data (such as comments and letters) were excluded. We manually reviewed the references included in each study to identify other relevant studies.

### 2.3 Assessment of study quality

The quality of all included studies was evaluated using the Newcastle–Ottawa Scale (NOS), in which a score of 1-9 stars was allocated. The more stars a study acquired, the higher quality it was.

### 2.4 Statistical analysis

The relevant data about oncological results was extracted from the included study, including local recurrence, metastasis, all-cause death, and cancer-specific death. Recurrence was defined as ipsilateral local renal or retroperitoneal recurrence according to imaging tests, including lymph nodes, resection bed and renal scar.

The meta-analysis was conducted using Review Manager Version 5.3 and Stata 14.2. Continuous variables were presented as weighted mean differences (WMDs) with 95% confidence intervals (CIs), and dichotomous variables were presented as odds ratios (ORs) with 95% CIs. If no significant heterogeneity between two groups was found, the fixed-effect model was used. Otherwise, the random-effect model was used. Furthermore, subgroup analyses were carried out to exclude the bias of different average follow-ups, which were divided into three subgroups: less than 3 years, between 3 and 6 years, and more than 6 years.

For studies reported as a median follow-up, it will be converted to an average according to the conversion formula (10). Heterogeneity among the studies was calculated using the χ2 test and I^2^ statistics. A p value < 0.10 and an I^2^ value >50% were considered to be significant. To evaluate publication bias, Egger’s test was performed.

## 3 Results

### 3.1 Study characteristics

**Figure 1:**
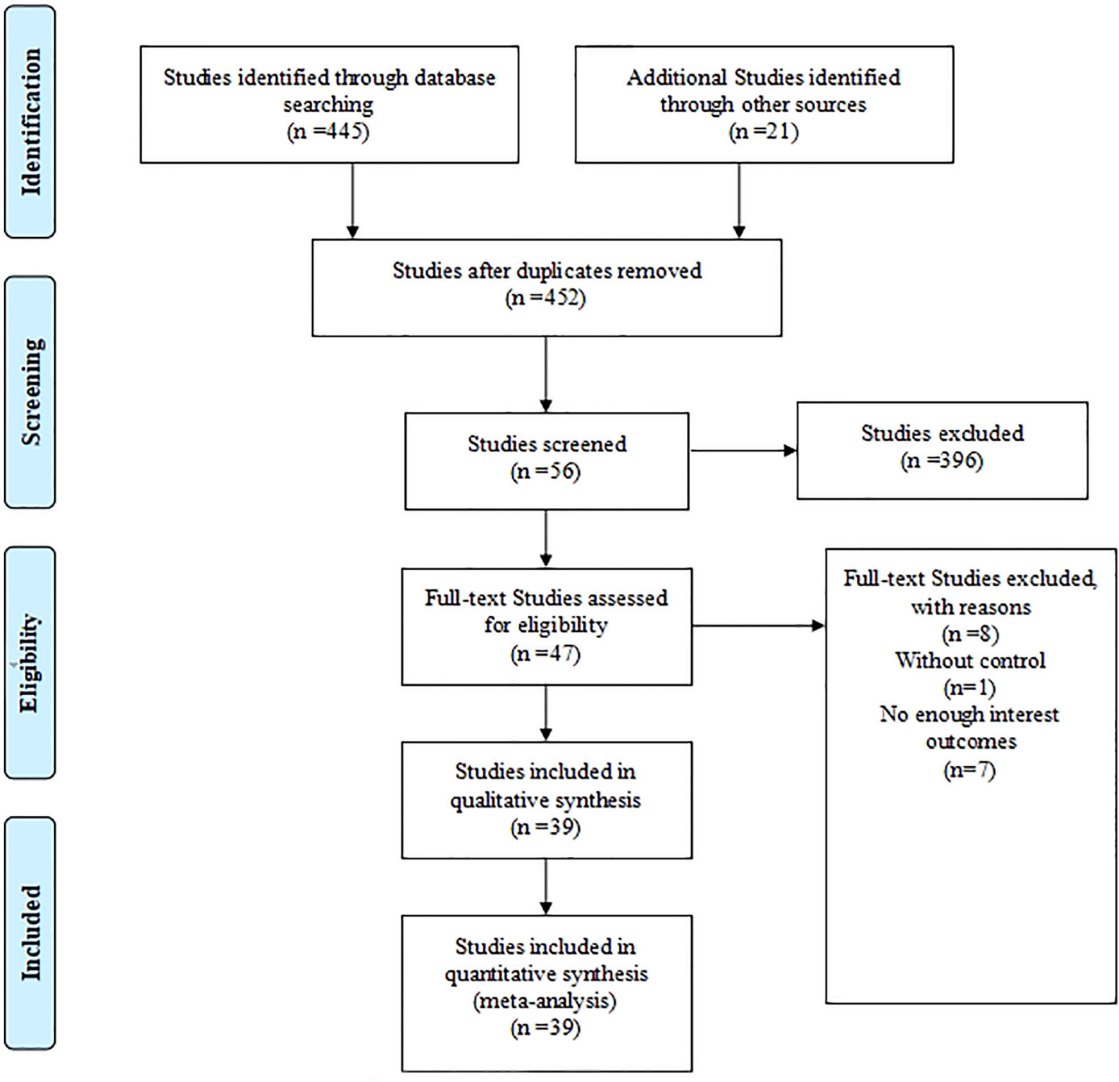
PRISMA flow diagram.

A total of 452 publications were searched after eliminating repetition, and 47 studies passed title screening. After identifying the abstracts and full texts according to the inclusion criteria, 8 studies were excluded (1 study had no control group, and 7 studies had no available results). The PRISMA flow diagram was presented in **Figure 1**.

Finally, 39 CCSs (4, 6–8, 11–45), with a total of 21461 patients were included, from which 1278 patients were assigned to the PSM group and 20183 cases to the NSM group. In addition, these studies were carried out from 13 countries in Europe, North America, South America, and Asia. The methods of operation included open, laparoscopic and robotic-assisted PN. The characteristics and quality of the included studies are summarized in **Table 1**. There were 9 studies (6, 16, 24, 27, 31–34, 44) explaining the specific quantitative differences between the two groups in different tumor stages. After statistical analysis, it was found that there were significant differences in patients with different stages between the two groups (p < 0.001) (**Table 2**).

**Table 1:** Characteristics and quality of included studies.

Reference	Study period	Histopathology (/N)	Design	Stage(/N)	Study origin	PSM/NSM	Surgical technique	Research project^▲^	Follow-up^a/b^(months)	SQ	FSA
***Favaretto*** ^11^	2000-2010	97-CCR, 27-PAR, 13-CHR	SC	120-pT1a,9-pT1b, 8-pT3a	France	2/135	Lap	1,4	38^b^	6	Yes
Costabel^12^	2003-2013	36-CCR, 4-PAR, 2-CHR	SC	NA	Argentina	4/41	Lap/Open	1	27.56^b^	6	Yes
Wang^13^	2003-2008	NA	SC	53-pT1	China	1/52	Lap	1,2	39^a^	6	No
Kara^14^	2011-2016	83-CCR, 13-PAR, 13-CHR, 12-OTR	SC	83-T1a, 13-T1b, 1-T2a, 24-T3a	USA	8/113	Robotic/Open	1,2	16.3^a^	6	No
Khalifeh^15^	2007-2012	578-CCR,223-PAR, 81-CHR, 61-OTR	MC	763-T1a, 107-T1b, 15-T2a, 1-T2b, 45-T3a	USA	21/922	Robotic	1,2	17.3^a^	7	Yes
***Rothberg^16^***	NA	530-CCR,158-PAR, 53-CHR, 98-OTR	MC	PSM:33-cT1a,8-cT1b,1-cT2a NSM:586-cT1a,196-cT1b,15-cT2a	USA	42/797	Robotic	3	18.8^a^	8	No
***Rothberg*** ^17^	2008-2017	298-CCR,89-PAR, 30-CHR, 15-OTR	SC	348-T1a, 57-T1b, 3-T2a, 19-T3a	USA	29/403	Robotic	1,2,3	45.1^a^	6	No
***Tiu*** ^18^	2009-2010	NA	SC	26-pT1a, 6-pT1b, 1-pT2	Korea	1/32	Robotic	1,2,3,4	26^b^	6	Yes
Maddox^20^	2008-2013	28-CCR, 6-PAR, 1-CHR	SC	32-pT1b, 1-pT2, 2-pT3	USA	3/32	Robotic/Open	1,2	24.3^b^	6	No
Kaczmarek^21^	2010-2011	NA	SC	NA	USA	3/142	Robotic	1	18^a^	6	No
***Ani*** ^24^	1995-2004	NA	MC	PSM:37-pT1a,15-pT1b, 5-pT2, 13-pT3a, 1-pT3b NSM:487-pT1a,55-pT1b,16-pT2, 33-pT3a, 2-pT3b	Canada	71/593	Lap/Open	3,4	94.8^b^	8	No
Permpongkosol^25^	1994-2005	NA	SC	NA	USA	9/502	Lap	1,2	18^a^	6	Yes
***Raz*** ^8^	1995-2005	NA	SC	NA	Israel	17/95	Lap/Open	3,4	71^b^	7	Yes
Yossepowitch^26^	1972-2005	880-CCR, 343-PAR, 130-CHR, 37-OTR	MC	1045-T1a,237-T1b, 27-T2, 79-T3	USA	77/1267	Lap/Open	1	40.8^b^	8	Yes
Wood^28^	2000-2014	NA	SC	140-T1a, 46-T1b, 3-T2, 17-T3	USA	12/193	Lap/Open	1	23^b^	6	No
Antic^29^	2005-2012	243-CCR,77-PAR, 47-CHR, 39-OTR	SC	299-T1a, 64-T1b, 14-T2, 29-T3	USA	61/345	Lap/Open	1	33.1^a^	6	No
Kang^30^	1999-2011	NA	MC	1581-T1a, 139-T1b	Korea	31/1782	Lap/Open	1,2	32.5^b^	8	Yes
***Kızılay*** ^4^	2015-2017	83-CCR, 24-PAR, 13-CHR, 5-OTR	SC	NA	Turkey	15/110	Lap/Open/ Robotic	1,2,3	55.35^a^	8	No
***Petros*** ^31^	1990-2015	NA	SC	PSM:23-T1a, 4-T1b, 7-T3a NSM:75-T1a, 17-T1b, 8-T3a	USA	34/100	Lap/Open/ Robotic	3	62^a^	8	Yes
***Bensalah*** ^32^	1987-2006	135-CCR,52-PAR, 12-CHR	MC,PM	PSM:93-T1,4-T2, 14-T3 NSM:598-T1, 21-T2, 45-T3	*Europe/ North America	101/102	Lap/Open/ Robotic	1,3,4	37^a^	8	No
Kwon^33^	1989-2005	NA	SC	PSM:44-T1a, 5-T1b, 1-T2,5-T3a/b NSM::528-T1a, 79-T1b, 8-T2, 54-T3a/b	USA	57/713	Lap/Open/ Robotic	1,2	22^b^	8	No
Shah^34^	2006-2013	NA	MC	PSM:86-cT1,2-cT2,9-cT3 NSM:1059-cT1,30-cT2,54-cT3	USA	97/1143	Lap/Open/ Robotic	1,2	33^b^	8	No
Tellini^6^	1983-2014	337-CCR,79-PAR, 31-CHR, 12-OTR	SC	PSM:21-pT1a,3-pT1b, 1-pT2,2-pT3a NSM:349-pT1a,61-pT1b, 6-pT2, 16-pT3a	Italy	27/432	Lap/Open	1,2	96^b^	8	No
Li^35^	2007-2017	NA	MC	NA	China	20/580	Lap/Open	1,2	56^b^	6	No
Eroglu^36^	2002-2003	NA	SC	12-pT1a, 6-pT1b	Turkey	3/15	Open	1,2	18^a^	6	Yes
Coffifin^37^	1980-2005	125-CCR,29-PAR, 1-CHR	SC	93-pT1a, 41-pT1b, 13-pT2,7-pT3a	France	15/140	Open	1	118.2^a^,95^b^	6	No
Desai^38^	2000-2007	NA	SC	49-T1a, 1-T1b	USA	5/45	Lap	1,2	43.7^a^	6	Yes
***Maurice*** ^39^	2003-2006	2044-CCR935-PAR, 68-CHR, 2691-OTR	MC	5773-T1, 125-T2, 140-T3a	USA	302/5736	Lap/Open	3	71^b^	7	No
Sutherland^40^	1988-1999	35-CCR, 8-PAR, 1-CHR	SC	42-pT1,2-pT2,1-pT3a	USA	3/41	Lap/Open	1,2	49^a^	6	No
***Saranchuk*** ^41^	1989-2003	NA	SC	NA	USA	8/46	Lap	1,2,4	40.5^a^,32.6^b^	6	No
Zigeuner^42^	1974-2000	93-CCR, 12-PAR, 3-CHR, 9-OTR	SC	90-pT1,6-pT2, 18-pT3,	Austria	6/108	Open	1,2	80^a^,69^b^	6	No
Bernhard^23^	1984-2006	569-CCR,160-PAR, 48-CHR	MC	591-T1a, 126-T1b, 28-T2, 64-T3a	USA/ France	12/797	Open	1	39^a^	6	No
Porpiglia^22^	1998-2004	60-CCR, 4-PAR	SC	61-pT1a, 2-pT1b, 1-pT3a	Italy	2/62	Lap/Open	1	31.9^a^	6	Yes
Rogers^19^	2002-2007	44-CCR, 21-PAR, 2-CHR, 42-OTR	MC	87-pT1a, 15-pT1b, 3-pT2,4-pT3a	USA	6/103	Robotic	1	7.2^a^	6	No
***López-Costea*** ^7^	1990-2004	137-CCR,35-PAR, 19-CHR, 6-OTR	SC	156-pT1a,37-pT1b, 1-pT2a, 4-pT3a	Spain	27/171	Lap/Open	3	56.1^a^	6	No
***Bansal*** ^27^	2011-2014	686-CCR,191-PAR, 71-CHR, 95-OTR	MC	PSM:43-pT1a,10-pT1b,1-pT2, 12-pT3 NSM:651-pT1a,175-pT1b,33-pT2, 54-pT3	Canada	68/935	Lap/Open/ Robotic	1,2,3,4	19^b^	8	No
Marchiñena^43^	2010-2015	245-CCR,25-PAR, 45-CHR,4-OTR	SC	266-pT1a,48-pT1b	Argentina	22/292	Lap/Open/ Robotic	1,2	24^b^	8	No
***Wahba*** ^44^	2007-2012	278-CCR,64-PAR, 32-OTR	SC	PSM:10-pT1a,2-pT1b,0-pT2 NSM:308-pT1a,44-pT1b,10-pT2	USA	12/362	Robotic	1,3	77.7^a^	8	No
Lee^45^	2005-2014	648-CCR,55-PAR,43-CHR 2-OTR	SC	658-T1a,90-Other stages	Korea	44/704	Lap/Open/ Robotic	1	58^b^	7	No

SQ, study quality according to the Newcastle–Ottawa scale; MC, Multiple center; SC, single center; PM, pair matched; NA, not available; Lap, laparoscopic; CCR, clear cell RCC; PAR, papillary RCC; CHR, chromophobe RCC; RCC, renal cell carcinoma; OTR, other RCC; Follow-upa/b, Follow-upmean/median; FSA, frozen section assessment.

^*^Including the countries France/Italy/Belgium/Israel/Germany/Spain/USA/Canada.

▲Research project: 1 = Local recurrence; 2 = Metastasis; 3 = All-cause death; 4 = Cancer specific death.

The authors of the study evaluated data on all-cause death or cancer-specific death, marking their names in italics in tables.

**Table 2:** Data of different tumor stages in 9 studies.

Stage	PSM	NSM	Total (N)	P
T1a	390	4641	5031	
T1b	47	627	674	
T2	15	139	154	
T3	63	266	329	
Total (N)	515	5673	–	<0.001

### 3.2 Outcomes for meta-analysis

#### 3.2.1 Local recurrence

**Figure 2:**
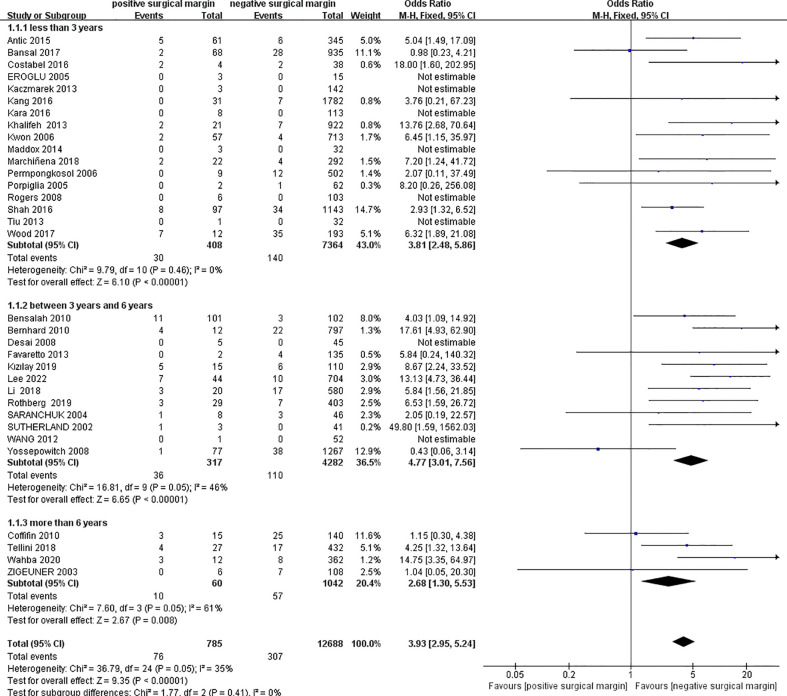
Comparison of local recurrence between positive surgical margin (PSM) and negative surgical margin (NSM) groups.

We extracted data on local recurrence in two groups from 33 studies (4, 6, 11–15, 17–23, 25–30, 32–38, 40–45) including 13473 patients (PSM: 785, NSM: 12688). In 8 studies (13, 14, 18–21, 36, 38), the OR value could not be calculated because the number of local recurrence was zero in both the PSM and NSM groups. The pooled results showed that the rate of local recurrence in the PSM group was significantly higher than that in the NSM group (OR 3.93, 95% CI 2.95-5.24; p < 0.00001). Egger’s test showed no published bias (P = 0.266). The results of subgroup analysis showed that the results of each subgroup were similar to the total results (**Figure 2**).

#### 3.2.2 Metastasis

**Figure 3:**
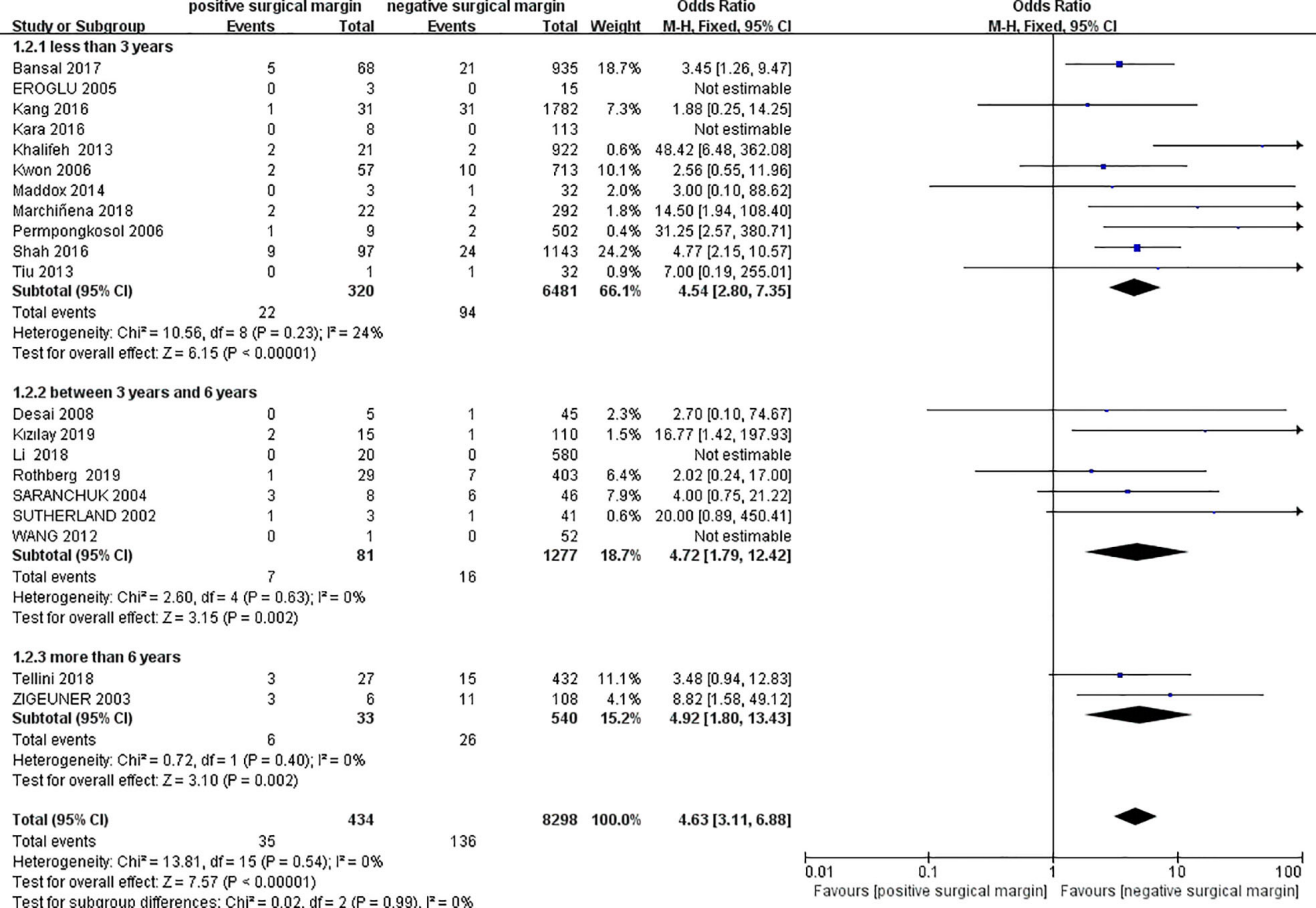
Comparison of metastasis between PSM and NSM groups.

Twenty studies provided comparative data on tumor metastasis between the two groups (4, 6, 13–15, 17, 18, 20, 25, 27, 30, 33–36, 38, 40–43), including 434 patients in the PSM group and 8298 patients in the NSM group. In 4 studies (13, 14, 35, 36), the number of metastatic cases in the two groups was zero, and the OR value could not be calculated. Pooled data showed a significantly higher rate of metastasis in the PSM group (OR 4.63, 95% CI 3.11-6.88; p < 0.00001), and similar results could be observed in subgroup analyses (**Figure 3**). Egger’s test showed insignificant published bias (P = 0.145).

#### 3.2.3 All-cause death

**Figure 4:**
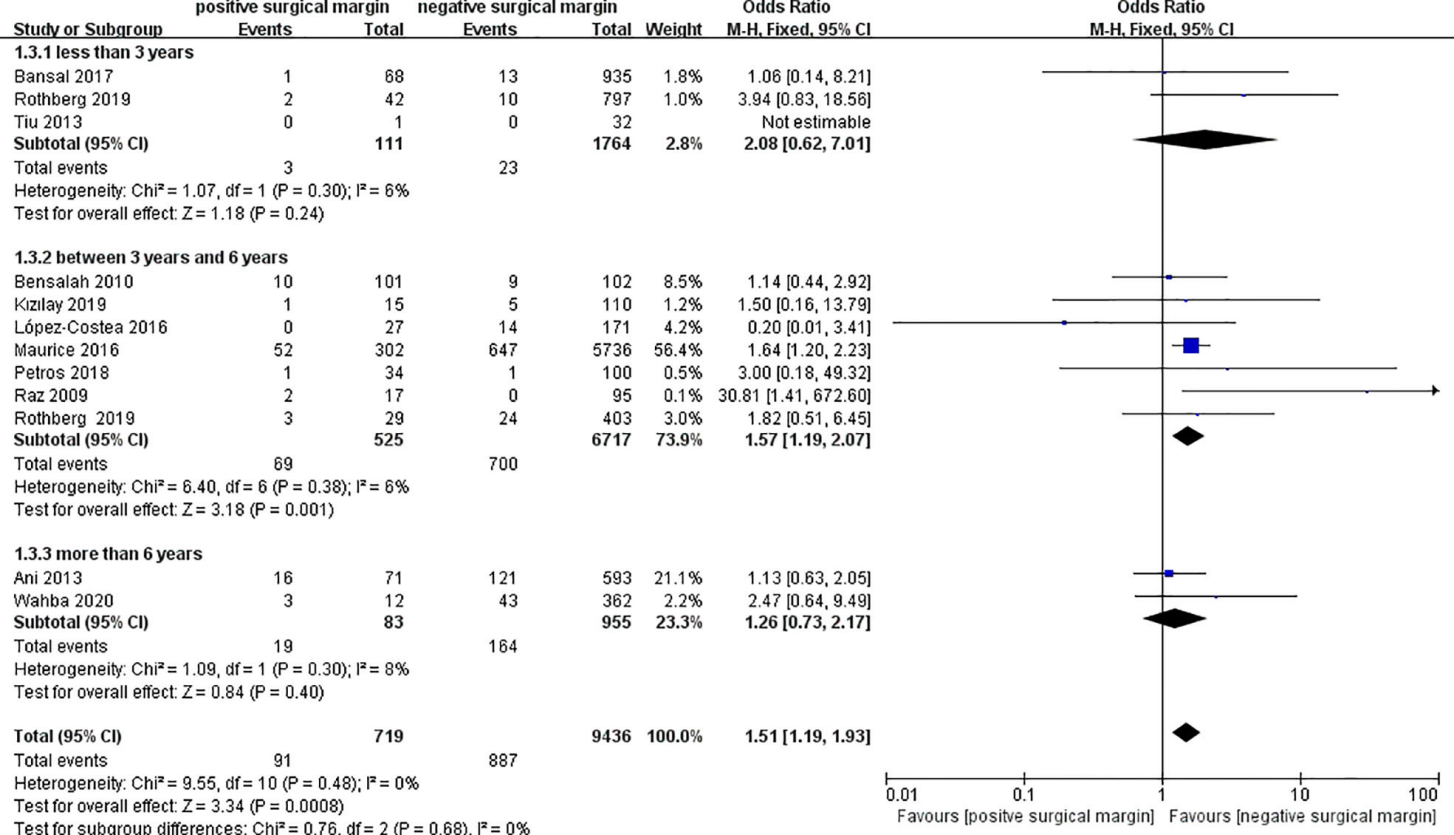
Comparison of all-cause death between PSM and NSM groups.

Twelve studies including 10155 patients reported data on all-cause death in two groups (4, 7, 8, 16–18, 24, 25, 31, 32, 39, 44), from which a significantly higher rate of all-cause death in the PSM group could be found than that in the NSM group (OR 1.51, 95% CI 1.19-1.93; p = 0.0008; **Figure 4**). However, subgroup analyses showed insignificant results between the two groups for those less than 3 years (OR 2.08, 95% CI 0.62-7.01; p = 0.24) and those more than 6 years (OR 1.26, 95% CI 0.73-2.17; p = 0.40) (**Figure 4**).

**Figure 5:**
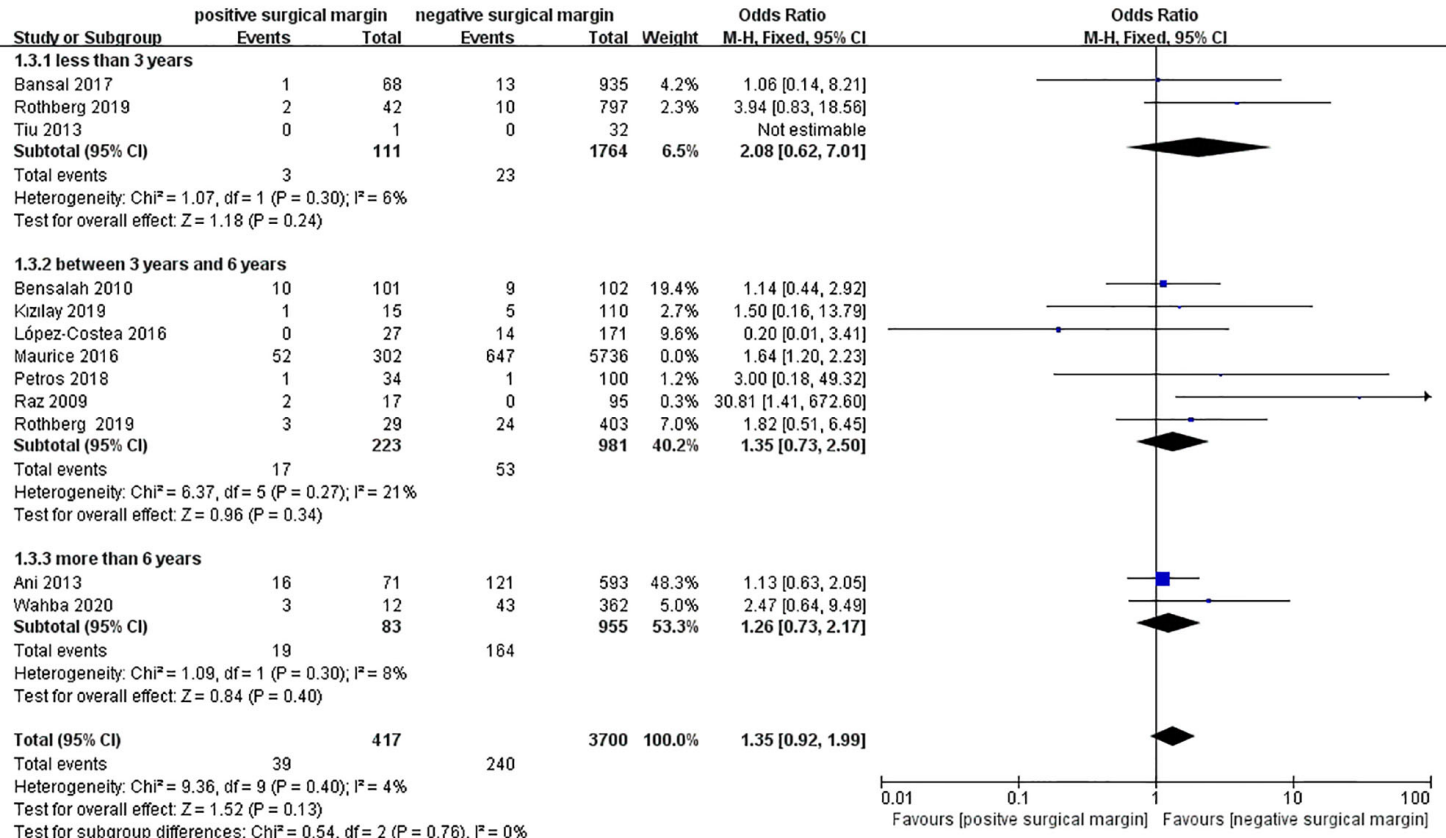
Sensitivity analysis of comparison of all-cause death between PSM and NSM groups.

Maurice et al. (39) performed a data analysis based on the National Cancer Database, which accounted for too much weight (57.6%). Therefore, a sensitivity analysis excluding this study to avoid possible duplication in the population was performed to test the credibility of the results. The final results after excluding Maurice’s study showed that there were no significant differences in the rate of all-cause death between the two groups in the subgroup analyses and pooled analysis (OR 1.35, 95% CI 0.92-1.99; p = 0.13) (**Figure 5**). Egger’s test showed no significant published bias (P = 0.209).

#### 3.2.4 Cancer-specific death

**Figure 6:**
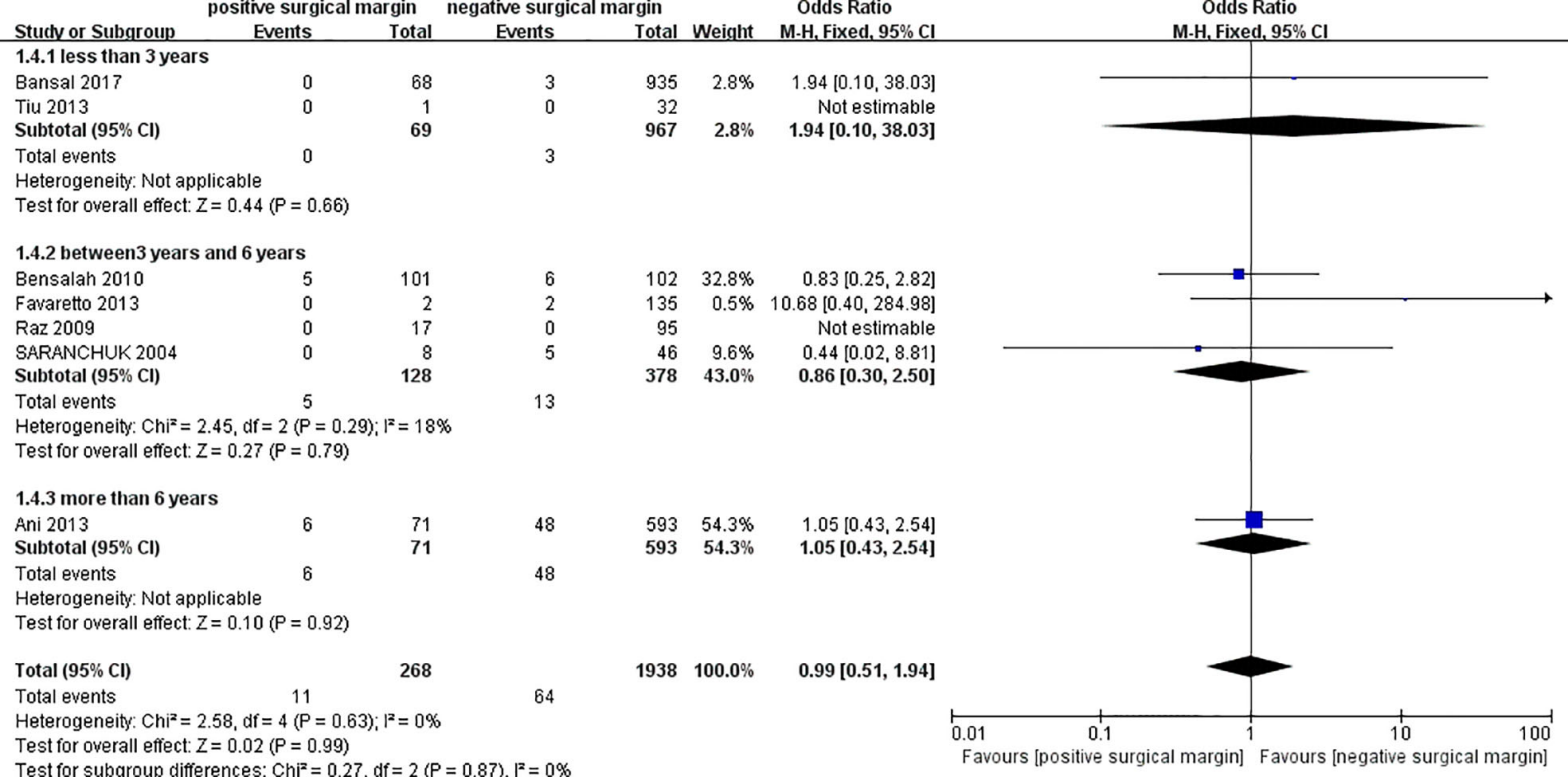
Comparison of cancer specific death between PSM and NSM groups.

Seven studies provided data on cancer-specific death in the two groups (8, 11, 18, 24, 27, 32, 41), which showed an insignificant difference in the incidence of cancer-specific death between the two groups (less than 3 years: OR 1.94, 95% CI 0.10-38.03; p = 0.66; between 3 years and 6 years: OR 0.86, 95% CI 0.30-2.50; p = 0.79; more than 6 years: OR 1.05, 95% CI 0.43-2.54; p = 0.92; and totally: OR 0.99, 95% CI 0.51-1.94; p = 0.99;, respectively) (**Figure 6**).

## 4 Discussion

RCC is a common malignancy in the urinary system, and its incidence is increasing year by year (46). PN is an internationally recognized standard treatment for localized renal tumors according to the EAU and AUA guidelines (1, 47). Although it can prevent the loss of renal function to some extent compared to radical nephrectomy, there is also a risk of incomplete resection of tumors, leading to PSM. It has been confirmed that NSM is very important in reducing the risk of tumor recurrence in many cancers, such as prostate cancer, bladder cancer, breast cancer, colon cancer, and pancreatic cancer (48–50). However, the effect of PSM of RCC on oncological outcomes after PN is still controversial.

The results of this study showed that there was a significant correlation between PSM and local recurrence and metastasis after PN, which was consistent with the results from Ficarra et al., in which a systematic review including 14 studies was conducted (51). Our study included more literature and carried out subgroup analyses, which might provide more support for these results. Moreover, Khalifeh et al. found that only PSM was statistically significant in tumor recurrence and metastasis (15). In a study by Memorial-Sloan Kettering Cancer Center, it was reported that renal tumors with high malignant potential were more likely to relapse when they were PSM (33). When PSM patients were subdivided into a low-risk group and a high-risk group according to the pathological stage and grade, it was found that PSM accompanied by high-risk pathology could significantly increase the risk of 5-year recurrence (34). In addition, other studies suggested recurrence is usually associated with patients at risk of multifocal tumors, in which recurrence might be due to new primary tumors rather than PSM (31). We speculated that the follow-up of patients with PSM might be more rigorous, which would also affect their prognosis.

Our study demonstrated that the rate of PSM could be calculated to be 6.36%, which was similar to 2%-8% (5) in previous publications. Multiple risk factors might have contributed to PSM, including age of patients (52), tumor location (53), tumor size[26 (24),], tumor stage (53), tumor grade (32, 52), tumor infiltration into perirenal fat (53), preoperative renal function (24), surgery volume of surgeons (54), surgical approach (9), etc.

The debate on how to further manage patients with PSM after PN has not been eliminated. Several measures/interventions could be adopted, including radical nephrectomy, tumor bed reresection, marginal energy ablation and observation. However, our results showed that there was an insignificant relationship between the two groups in all-cause death and cancer-specific death, which demonstrated that vigilant and close monitoring might be an effective strategy in lieu of active intervention (55).

It should be carefully noted that the 5-year survival of localized RCC was up to 75% but less than 10% for metastatic RCC (56). In our study, PSM had a more than fourfold impact on the metastasis of RCC, but an impact on the survival of patients was not found. This might be related to insufficient follow-up duration. Although subgroup analyses were carried out, the follow-up of most studies was less than 5 years. In addition, according to the latest cancer report in 2021 (57), it was mentioned that there were some differences in the survival for patients with different pathological types of RCC in different populations, which was not separately analyzed in our study. In this study, it was found that there was a significant difference in tumor stage between the PSM group and the NSM group, although the data of this analysis only came from 9 studies. This still reminded us that tumor stage might also have an impact on the results of the study. Meanwhile, a few studies (8, 11, 12, 15, 18, 22, 25, 26, 30, 31, 36, 38) have mentioned the use of frozen section assessment (FSA) in surgery. However, Miyamoto’s (58) study suggested that the diagnostic accuracy of FSA in partial nephrectomy and its impact on the status of the incision margin had not been determined. They did not believe that FSA will benefit patients. In their opinion, there remains limitations in the FSA diagnosis on margin specimens, such as inadequate sampling and suboptimal tissue preparation due to histologic frozen artifact or cautery artifact, any of which may result in an indeterminate or inaccurate interpretation. Indeed, the diagnostic accuracy of FSA at the surgical margins is not necessarily high, while it is dependent on the pathologist’s knowledge and experience. This issue may require further RCT to determine the usefulness of FSA on surgical margins.

Above all, several potential limitations of this meta-analysis must be taken into consideration. Because all the included studies were retrospective CCSs, the quality of this study was limited. In addition, the heterogeneity among the studies could be easily affected by different urologists’ experience, surgical technique, patient selection, tumor size, stage, grade and location, and incompletely uniform follow-up, which could cause possible biases. Moreover, different methods and their combinations, including open, laparoscopy and robot-assisted laparoscopy, were used in these studies, which made it difficult to carry out subgroup analyses.

## Conclusion

By comparing the effects of different surgical margin states on the development of RCC after PN, we found that PSM was closely related to postoperative tumor recurrence and metastasis. However, there was no significant difference in all-cause death or cancer-specific death between the PSM and NSM groups. Therefore, for patients with PSM after PN, close monitoring might be another effective choice apart from active treatments. Considering some limitations in our study, we strongly suggest that our results be carefully cautioned and further validated.

## Author contributions

RB, LG, JW and QJ conceived and designed the study. RB and LG performed the analysis, prepared the figures and tables, and wrote the main manuscript. All of the authors reviewed the manuscript. All authors read and approved the final manuscript.

## Conflict of interest

The authors declare that the research was conducted in the absence of any commercial or financial relationships that could be construed as a potential conflict of interest.

## Publisher’s note

All claims expressed in this article are solely those of the authors and do not necessarily represent those of their affiliated organizations, or those of the publisher, the editors and the reviewers. Any product that may be evaluated in this article, or claim that may be made by its manufacturer, is not guaranteed or endorsed by the publisher.

## References

[1] Ljungberg B Albiges L Abu-Ghanem Y Bensalah K Dabestani S Fernández-Pello S. European Association of urology guidelines on renal cell carcinoma: The 2019 update. Eur Urol. (2019) 75(5):799–810. doi: 10.1016/j.eururo.2019.02.011 30803729

[2] Crépel M Jeldres C Sun M Lughezzani G Isbarn H Alasker A. A population-based comparison of cancer-control rates between radical and partial nephrectomy for T1A renal cell carcinoma. Urology (2010) 76(4):883–8. doi: 10.1016/j.urology.2009.08.028 20932408

[3] Deklaj T Lifshitz DA Shikanov SA Katz MH Zorn KC Shalhav AL. Laparoscopic radical versus laparoscopic partial nephrectomy for clinical T1b N0 M0 renal tumors: Comparison of perioperative, pathological, and functional outcomes. J Endourol. (2010) 24(10):1603–7. doi: 10.1089/end.2009.0312 20932215

[4] Kizilay F Eskidemir U Bahceci T Simsir A Ozdemir H Sarsik B. The effect of surgical margin on cancer-specific survival in patients treated with nephron-sparing surgery. Niger J Clin Pract (2019) 22(10):1396. doi: 10.4103/njcp.njcp_267_18 31607729

[5] Choi JE You JH Kim DK Rha KH Lee SH. Comparison of perioperative outcomes between robotic and laparoscopic partial nephrectomy: A systematic review and meta-analysis. Eur Urol. (2015) 67(5):891–901. doi: 10.1016/j.eururo.2014.12.028 25572825

[6] Tellini R Antonelli A Tardanico R Fisogni S Veccia A Furlan MC. Positive surgical margins predict progression-free survival after nephron-sparing surgery for renal cell carcinoma: Results from a single center cohort of 459 cases with a minimum follow-up of 5 years. Clin Genitourin Canc. (2019) 17(1):e26–31. doi: 10.1016/j.clgc.2018.08.004 30266249

[7] López-Costea MÁ Bonet X Pérez-Reggeti J Etcheverry B Vigués F. Oncological outcomes and prognostic factors after nephron-sparing surgery in renal cell carcinoma. Int UROL Nephrol. (2016) 48(5):681–6. doi: 10.1007/s11255-016-1217-z 26861062

[8] Raz O Mendlovic S Shilo Y Leibovici D Sandbank J Lindner A. Positive surgical margins with renal cell carcinoma have a limited influence on long-term oncological outcomes of nephron sparing surgery. Urology. (2010) 75(2):277–80. doi: 10.1016/j.urology.2009.06.110 19896179

[9] Tabayoyong W Abouassaly R Kiechle JE Cherullo EE Meropol NJ Shah ND. Variation in surgical margin status by surgical approach among patients undergoing partial nephrectomy for small renal masses. J Urol (2015) 194(6):1548–53. doi: 10.1016/j.juro.2015.06.076 26094808

[10] Hozo SP Djulbegovic B Hozo I. Estimating the mean and variance from the median, range, and the size of a sample. BMC Med Res Methodol (2005) 5(1):5–13. doi: 10.1186/1471-2288-5-13 PMC1097734 15840177

[11] Favaretto RL Sanchez-Salas R Benoist N Ercolani M Forgues A Galiano M. Oncologic outcomes after laparoscopic partial nephrectomy: Mid-term results. J Endourol. (2013) 27(1):52–7. doi: 10.1089/end.2012.0132 22788241

[12] Costabel JI Marchiñena PG Tirapegui F Dantur A Jurado A Gueglio G. Functional and oncologic outcomes after nephron-sparing surgery in a solitary kidney: 10 years of experience. Int Braz J Urol. (2016) 42(2):253–61. doi: 10.1590/S1677-5538.IBJU.2014.0463 PMC4871385 27256179

[13] Wang J Qi L Zu X Chen M. Application of retroperitoneal laparoscopic partial nephrectomy for renal cell carcinoma of the early stage. Zhong Nan Da Xue Xue Bao. Yi Xue Ban = J Of Cent South University. Med Sci (2012) 37(5):485. doi: 10.3969/j.issn.1672-7347.2012.05.010 22659661

[14] Kara O Maurice MJ Malkoc E Ramirez D Nelson RJ Caputo PA. Comparison of robot-assisted and open partial nephrectomy for completely endophytic renal tumours: a single centre experience. Bju Int (2016) 118(6):946–51. doi: 10.1111/bju.13572 27477777

[15] Khalifeh A Kaouk JH Bhayani S Rogers C Stifelman M Tanagho YS. Positive surgical margins in robot-assisted partial nephrectomy: A multi-institutional analysis of oncologic outcomes (Leave no tumor behind). J Urol (2013) 190(5):1674–9. doi: 10.1016/j.juro.2013.05.110 23764077

[16] Rothberg MB Paulucci DJ Okhawere KE Reynolds CR Badani KK Abaza R. A multi-institutional analysis of the effect of positive surgical margins following robot-assisted partial nephrectomy on oncologic outcomes. J ENDOUROL. (2020) 34(3):304–11. doi: 10.1089/end.2019.0506 31931607

[17] Rothberg MB Peak TC Reynolds CR Hemal AK. Long-term oncologic outcomes of positive surgical margins following robot-assisted partial nephrectomy. Trans Androl Urol (2020) 9(2):879–86. doi: 10.21037/tau.2019.11.21 PMC7214993 32420203

[18] Tiu A Shin TY Kim KH Lim SK Han WK Rha KH. Robotic laparoendoscopic single-site transumbilical partial nephrectomy: Functional and oncologic outcomes at 2 years. Urology. (2013) 82(3):595–9. doi: 10.1016/j.urology.2013.05.010 23890663

[19] Rogers CG Menon M Weise ES Gettman MT Frank I Shephard DL. Robotic partial nephrectomy: a multi-institutional analysis. J Robotic Surg (2008) 2(3):141–3. doi: 10.1007/s11701-008-0098-2 27628250

[20] Maddox M Mandava S Liu J Boonjindasup A Lee BR. Robotic partial nephrectomy for clinical stage T1b tumors: Intermediate oncologic and functional outcomes. Clin Genitourin Canc. (2015) 13(1):94–9. doi: 10.1016/j.clgc.2014.07.011 25176501

[21] Kaczmarek BF Sukumar S Kumar RK Desa N Jost K Diaz M. Comparison of robotic and laparoscopic ultrasound probes for robotic partial nephrectomy. J Endourol. (2013) 27(9):1137–40. doi: 10.1089/end.2012.0528 23510382

[22] Porpiglia F Fiori C Terrone C Bollito E Fontana D Scarpa Rm. Assessment of surgical margins in renal cell carcinoma after nephron sparing: A comparative study. J Urol (2005) 173(4):1098–101. doi: 10.1097/01.ju.0000148360.47191.5e 15758709

[23] Bernhard J Pantuck AJ Wallerand H Crepel M Ferrière J Bellec L. Predictive factors for ipsilateral recurrence after nephron-sparing surgery in renal cell carcinoma. Eur Urol. (2010) 57(6):1080–6. doi: 10.1016/j.eururo.2010.02.019 20188458

[24] Ani I Finelli A Alibhai SMH Timilshina N Fleshner N Abouassaly R. Prevalence and impact on survival of positive surgical margins in partial nephrectomy for renal cell carcinoma: a population-based study. Bju Int (2013) 111(8):E300–5. doi: 10.1111/j.1464-410X.2012.11675.x 23305148

[25] Permpongkosol S Colombo JR Gill IS Kavoussi LR. Positive surgical parenchymal margin after laparoscopic partial nephrectomy for renal cell carcinoma: Oncological outcomes. J Urol (2006) 176(6):2401–4. doi: 10.1016/j.juro.2006.08.008 17085113

[26] Yossepowitch O Thompson RH Leibovich BC Eggener SE Pettus JA Kwon ED. Positive surgical margins at partial nephrectomy: Predictors and oncological outcomes. J Urol (2008) 179(6):2158–63. doi: 10.1016/j.juro.2008.01.100 PMC2704565 18423758

[27] Bansal RK Tanguay S Finelli A Rendon R Moore RB Breau RH. Positive surgical margins during partial nephrectomy for renal cell carcinoma: Results from Canadian kidney cancer information system (Ckcis) collaborative. Can Urolog Assoc J (2017) 11(6):182–7. doi: 10.5489/cuaj.4264 PMC5472463 28652876

[28] Wood EL Adibi M Qiao W Brandt J Zhang M Tamboli P. Local tumor bed recurrence following partial nephrectomy in patients with small renal masses. J Urol (2018) 199(2):393–400. doi: 10.1016/j.juro.2017.09.072 28941919

[29] Antic T. Taxy JB. partial nephrectomy for renal tumors. Am J Clin Pathol (2015) 143(5):645–51. doi: 10.1309/AJCP7LKLZ8JSJQRG 25873497

[30] Kang HW Lee SK Kim WT Yun SJ Lee S Kim W. Surgical margin does not influence recurrence rate in Pt1 clear cell renal cell carcinoma after partial nephrectomy: A multicenter study. J Surg Oncol (2016) 114(1):70–4. doi: 10.1002/jso.24259 27074886

[31] Petros FG Metcalfe MJ Yu K Keskin SK Fellman BM Chang CM. Oncologic outcomes of patients with positive surgical margin after partial nephrectomy: a 25-year single institution experience. World J Urol. (2018) 36(7):1093–101. doi: 10.1007/s00345-018-2241-7 29488096

[32] Bensalah K Pantuck AJ Rioux-Leclercq N Thuret R Montorsi F Karakiewicz PI. Positive surgical margin appears to have negligible impact on survival of renal cell carcinomas treated by nephron-sparing surgery. Eur Urol. (2010) 57(3):466–73. doi: 10.1016/j.eururo.2009.03.048 19359089

[33] Kwon EO Carver BS Snyder ME Russo P. Impact of positive surgical margins in patients undergoing partial nephrectomy for renal cortical tumours. Bju Int (2007) 99(2):286–9. doi: 10.1111/j.1464-410X.2006.06623.x 17155985

[34] Shah PH Moreira DM Okhunov Z Patel VR Chopra S Razmaria AA. Positive surgical margins increase risk of recurrence after partial nephrectomy for high risk renal tumors. J Urol (2016) 196(2):327–34. doi: 10.1016/j.juro.2016.02.075 PMC9235535 26907508

[35] Li G Zhu D Lang Z Wang A Li Y Zhang R. Classification of positive surgical margins and tumor recurrence after nephron-sparing surgery for small renal masses. (2018) 10:6591–8. doi: 10.2147/CMAR.S181843 PMC6283258 30584355

[36] Eroğlu M Ünsal A Bakirtaş H Tekdoğan ÜT Ataoğlu Ö Balbay MD. Routine frozen-section biopsy from the surgical bed should be performed during nephron-sparing surgery for renal cell carcinoma. Scandinavian J Urol Nephrol (2009) 39(3):222–5. doi: 10.1080/00365590510007757 16118094

[37] Coffin G Hupertan V Taksin L Vaessen C Chartier-Kastler E Bitker M. Impact of elective versus imperative indications on oncologic outcomes after open nephron-sparing surgery for the treatment of sporadic renal cell carcinomas. Ann Surg Oncol (2011) 18(4):1151–7. doi: 10.1245/s10434-010-1457-6 21136181

[38] Desai PJ Andrews PE Ferrigni RG Castle EP. Laparoscopic partial nephrectomy at the Mayo clinic Arizona: Follow-up surveillance of positive margin disease. Urology. (2008) 71(2):283–6. doi: 10.1016/j.urology.2007.08.050 18308104

[39] Maurice MJ Zhu H Kim SP Abouassaly R. Reexamining the association between positive surgical margins and survival after partial nephrectomy in a Large American cohort. J Endourol. (2016) 30(6):698–703. doi: 10.1089/end.2016.0031 26888059

[40] Se S Mi R Gt M Hb G. Does the size of the surgical margin in partial nephrectomy for renal cell cancer really matter? J Urol (2002) 167(1):61–4. doi: 10.1016/S0022-5347(05)65383-9 11743276

[41] Saranchuk JW Touijer AK Hakimian P Snyder ME Russo P. Partial nephrectomy for patients with a solitary kidney: The memorial Sloan-Kettering experience. Bju Int (2004) 94(9):1323–8. doi: 10.1111/j.1464-410X.2004.05165.x 15610114

[42] Zigeuner R Quehenberger F Pummer K Petritsch P Hubmer G. Long-term results of nephron-sparing surgery for renal cell carcinoma in 114 patients: Risk factors for progressive disease. Bju Int (2003) 92(6):567–71. doi: 10.1046/j.1464-410X.2003.04414.x 14511035

[43] Marchiñena PG Tirapegui S Gonzalez IT Jurado A Gueglio G. Positive surgical margins are predictors of local recurrence in conservative kidney surgery for Pt1 tumors. Int Braz J Urol. (2018) 44(3):475–82. doi: 10.1590/s1677-5538.ibju.2017.0039 PMC5996790 29368873

[44] Wahba BM Chow AK Du K Sands KG Paradis AG Vetter JM. Positive surgical margins after robot-assisted partial nephrectomy predict long-term oncologic outcomes for clinically localized renal masses. J Endourol (2021). doi: 10.1089/end.2020.0707 PMC8252897 33267669

[45] Lee J Kim J Kim JC Ham WS Han WK Rha KH. Evaluation of the surgical margin threshold for avoiding recurrence after partial nephrectomy in patients with renal cell carcinoma. Yonsei Med J (2022) 63(2):173–8. doi: 10.3349/ymj.2022.63.2.173 PMC8819404 35083903

[46] King SC Pollack LA Li J King JB Master VA. Continued increase in incidence of renal cell carcinoma, especially in young patients and high grade disease: United states 2001 to 2010. J Urol (2014) 191(6):1665–70. doi: 10.1016/j.juro.2013.12.046 PMC4479175 24423441

[47] Campbell S Uzzo RG Allaf ME Bass EB Cadeddu JA Chang A. Renal mass and localized renal cancer: AUA guideline. J Urol (2017) 198(3):520–9. doi: 10.1016/j.juro.2017.04.100 28479239

[48] Eastham Ja Kattan Mw Riedel E Begg Cb Wheeler Tm Gerigk C. Variations among individual surgeons in the rate of positive surgical margins in radical prostatectomy specimens. J Urol (2003) 170(6):2292–5. doi: 10.1097/01.ju.0000091100.83725.51 14634399

[49] Tseng JF Pisters PWT Lee JE Wang H Gomez HF Sun CC. The learning curve in pancreatic surgery. Surgery. (2007) 141(4):456–63. doi: 10.1016/j.surg.2006.09.013 17383522

[50] Swallow CJ Catton CN. Local management of adult soft tissue sarcomas. Semin Oncol (2007) 34(3):256–69. doi: 10.1053/j.seminoncol.2007.03.008 17560988

[51] Ficarra V Crestani A Inferrera A Novara G Rossanese M Subba E. Positive surgical margins after partial nephrectomy: A systematic review and meta-analysis of comparative studies. Kidney Cancer. (2018) 2(2):133–45. doi: 10.3233/KCA-180037

[52] Santarosa M Favaro D Quaia M Galligioni E. Expression of heat shock protein 72 in renal cell carcinoma: Possible role and prognostic implications in cancer patients. Eur J CANCER. (1997) 33(6):873–7. doi: 10.1016/S0959-8049(97)00002-6 9291808

[53] Schiavina R Serni S Mari A Antonelli A Bertolo R Bianchi G. A prospective, multicenter evaluation of predictive factors for positive surgical margins after nephron-sparing surgery for renal cell carcinoma: The RECORd1 Italian project. Clin Genitourin Canc. (2015) 13(2):165–70. doi: 10.1016/j.clgc.2014.08.008 25450033

[54] Malkoc E Maurice MJ Kara O Ramirez D Nelson RJ Dagenais J. Predictors of positive surgical margins in patients undergoing partial nephrectomy: A Large single-center experience. Türk Üroloji Dergisi/Turkish J Urol (2019) 45(1):17–21. doi: 10.5152/tud.2018.57767 PMC6342579 30668306

[55] Borghesi M Brunocilla E Schiavina R Martorana G. Positive surgical margins after nephron-sparing surgery for renal cell carcinoma: Incidence, clinical impact, and management. Clin Genitourin Canc. (2013) 11(1):5–9. doi: 10.1016/j.clgc.2012.09.010 23083800

[56] Zhang H Zhu G. Predictive biomarkers and updated targets of current guidance in treatment of metastatic renal cell carcinoma. Curr Med Chem (2020) 28:31–8. doi: 10.1097/CAD.0000000000000931 33357191

[57] Siegel RL Miller KD Fuchs HE Jemal A. Cancer statistics, 2021. CA: A Cancer J Clin (2021) 71(1):7–33. doi: 10.3322/caac.21654 33433946

[58] Miyamoto H. Intraoperative pathology consultation during urological surgery: Impact on final margin status and pitfalls of frozen section diagnosis. Pathol Int (2021) 71(9):567–80. doi: 10.1111/pin.13132 34154033

